# Application of artificial intelligence in esophageal surgery: a systematic review

**DOI:** 10.1007/s11701-025-02854-9

**Published:** 2025-10-16

**Authors:** Janosch Kröger, Nicolas Jorek, Alexander Seitel, Leon Mayer, Gabriel A. Salg, Nerma Crnovrsanin, Frank Pianka, Thomas Pausch, Lena Maier-Hein, Christoph Michalski, Henrik Nienhüser

**Affiliations:** 1 Department of General, Visceral and Transplantation Surgery, Heidelberg University Hospital, Heidelberg, Germany; 2 German Cancer Research Center (DKFZ), Division of Intelligent Medical Systems, Heidelberg, Germany; 3 National Center for Tumor Diseases (NCT), NCT Heidelberg, a partnership between DKFZ and University Hospital Heidelberg, Heidelberg, Germany; 4 Medical Faculty, Heidelberg University, Heidelberg, Germany; 5 Faculty of Mathematics and Computer Sciences, Heidelberg University, Heidelberg, Germany; 6 HIDSS4Health - Helmholtz Information and Data Science School for Health, KarlsruheHeidelberg, Germany

**Keywords:** Esophageal surgery, Artificial intelligence, Minimally invasive surgery, Machine learning

## Abstract

The aim of this systematic review was to summarize and analyze the available literature on the application of artificial intelligence systems in esophageal surgery, focusing on anatomy recognition, instrument detection, and surgical phase recognition. Esophageal cancer poses a significant global health challenge, ranking as the seventh most common cancer worldwide. Esophagectomy is the only curative treatment for non-metastatic esophageal cancer. While the introduction of minimally invasive esophagectomy and later robot-assisted minimally invasive esophagectomy significantly improved surgical precision and patient outcome, this development promoted a transition to increasing digitalization and video processing. Subsequently facilitating the integration of artificial intelligence is a promising tool in the enhancement of esophageal surgery. A systematic search was conducted following the PRISMA guidelines in the Medline and Web of Science databases. Studies published between January 2019 and June 2025 published in English and without restrictions to study type were included. Inclusion criteria focused on artificial intelligence-based anatomy recognition, instrument recognition, and phase recognition in esophageal surgery. Studies addressing preoperative and postoperative risk prediction or artificial intelligence applications not directly related to the surgical procedure were excluded. The systematic literature search yielded 7063 results. After screening, we identified six studies examining artificial intelligence applications in esophagectomy focusing on anatomy, instrument, and phase recognition. Artificial intelligence can be a useful tool—especially for intraoperative anatomy recognition—reaching detection rates comparable to trained surgeons in real time as seen in one study, reaching a Dice coefficient of 0.58, which was close to that of an expert esophageal surgeon (0.62) and significantly higher than the general surgeon (0.47, *p*= 0.0019). Due to the heterogeneity of study aims, utilized algorithms and outcome measures direct comparison between studies was not feasible. Artificial intelligence has demonstrated significant potential in enhancing esophageal surgery by improving anatomical recognition and optimizing surgical workflow. Despite these advancements, challenges remain in standardizing datasets, refinement of annotation methodologies, and seamless integration into real-time surgical navigation systems. To ensure clinical applicability, future research should focus on large-scale validation and prospective clinical trials to establish artificial intelligence’s clinical utility and safety in minimally invasive esophagectomy.

**Supplementary Information:**

The online version contains supplementary material available at 10.1007/s11701-025-02854-9.

## Introduction

Esophageal cancer represents a significant global health challenge, ranking as the seventh most common cancer worldwide in terms of incidence and the sixth leading cause of cancer-related mortality with esophagectomy being the only curative treatment in non-metastatic esophageal cancer [1, 2]. First successfully performed by Franz Torek in 1913, the procedure has seen incremental development over the past century [3]. A significant milestone was the introduction of minimally invasive esophagectomy (MIE), first described by Alfred Cuschieri in 1996 followed by robot-assisted minimally invasive esophagectomy (RAMIE) in 2003 [4, 5]. MIE has been demonstrated to enhance perioperative outcomes without compromising oncological results [6, 7]. With rising incidence of esophageal cancer and surgical resection being the only curative treatment, innovations in this field have the potential to improve patient outcome and assist the surgeon.

RAMIE offers three-dimensional visualization and full articulation of the surgical instruments. In addition, it allows for tremor filtering and motion scaling, thus enabling surgeons to perform precise manipulations, even in narrow surgical fields [8].

Challenges associated with MIE or RAMIE often arise from difficulties in identifying anatomical structures, particularly in cases where the surgeon is less experienced or when the operating field is compromised by bleeding or smoke [9–11]. Therefore, preventative strategies in avoiding intraoperative surgical complications are needed. Especially, the video recordings during minimally invasive procedures can be used for the implementation of various Artificial intelligence (AI) applications.

AI can be viewed as an overarching term that covers a wide range of data processing mechanisms. It refers to the use of algorithms to simulate human intelligence, including learning, reasoning, self-correction, and complex decision-making as seen in Machine learning (ML) for natural language recognition and processing or multimodal data analysis [12–14].

With respect to precise diagnostics, personalized medicine and workflow optimization, AI plays a promising role in making healthcare more efficient, especially in the surgical setting [15, 16]. In the operating room, AI holds great potential for enhancing surgical precision, enabling real-time decision-making, assisting in the identification of critical structures and improving patient safety. The number of studies exploring the implementation of AI in surgery is on the rise, with a focus on high-volume procedures such as cholecystectomy [17, 18]. Recent studies have demonstrated that procedures such as cholecystectomy can even be performed fully automated in ex-vivo experiments [19]. Concepts like anatomical structure, instrument, and phase recognition are central topics in many scientific papers. However, in more complex and less frequently performed surgeries, such as esophagectomy, the application of AI remains underexplored.

ML and deep learning (DL) have been employed to integrate AI in MIE in several published articles. The goal of this review is to assess and summarize the existing literature, focusing on their objectives, AI implementation and outcomes.

## Methods

### Search strategy and inclusion criteria

This systematic review was conducted in adherence to the PRISMA (Preferred Reporting Items for Systematic Reviews and Meta-Analyses) guidelines and as outlined in a predefined protocol (PROSPERO: CRD420251068871) [20].

To conduct this systematic review, we performed a comprehensive literature search to identify relevant articles exploring the intersection of esophagectomy and artificial intelligence applications. The analysis was conducted on articles published between January 2019 and June 2025. With most significant advancements in AI technology, particularly in deep learning and visual data analysis, occurring in recent years and to ensure the inclusion of the most recent publications, we decided on this timeframe.

The search was performed using the Medline and Web of Science databases, focusing on studies indexed under relevant Medical Subject Headings (MeSH). The applied search terms are listed in **Supplement 1**. The inclusion criteria emphasized studies that utilized AI for anatomical recognition, surgical phase detection, video analysis, and other computer-assisted methodologies in esophageal surgeries. Furthermore, only articles in English language and available in full text were included. Publications outside the timeframe of 2019–2025 or without substantial relevance to the combination of AI and esophagectomy were excluded. Currently ongoing trials were not considered for this review.

### Study selection

**Fig. 1:**
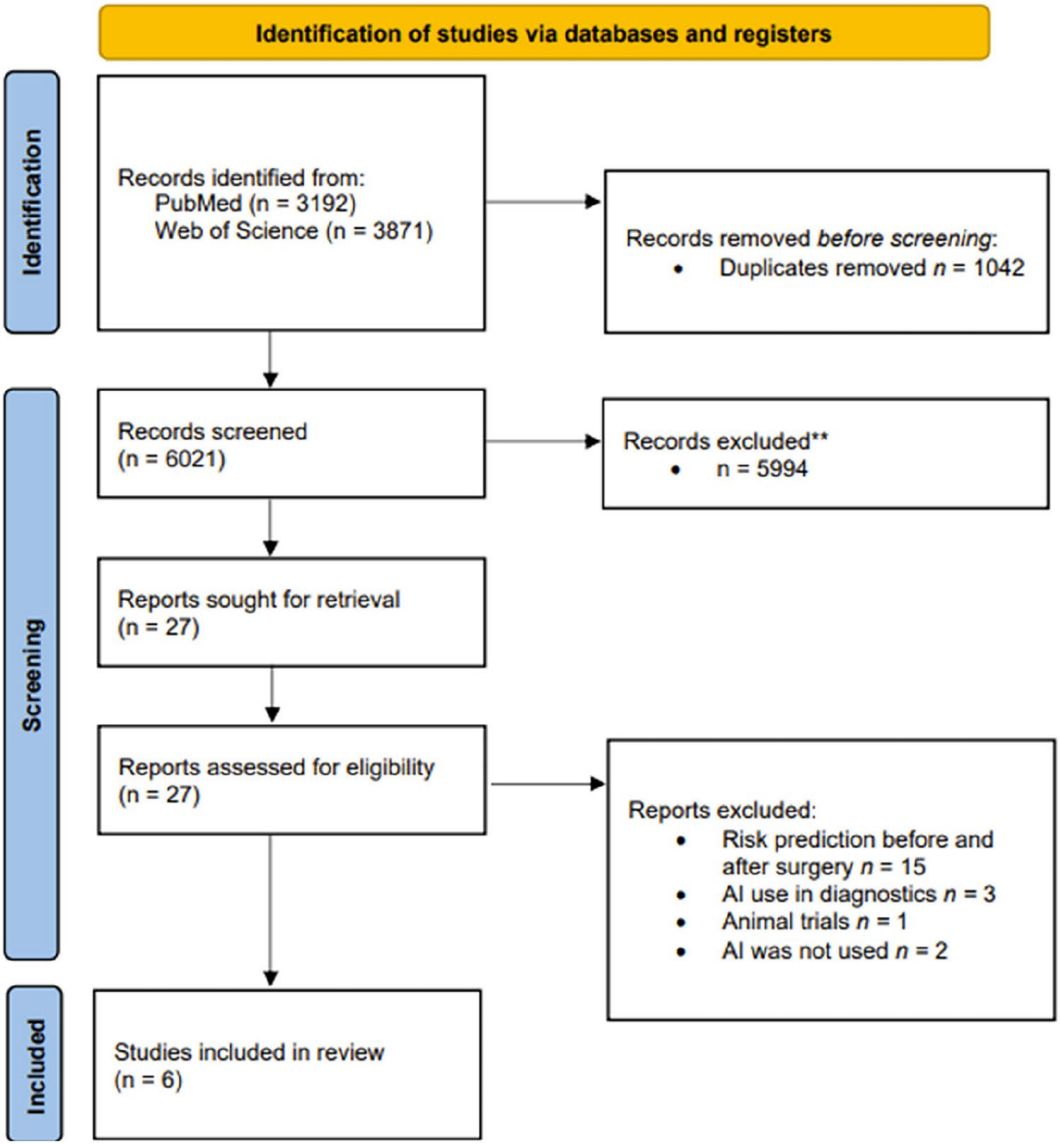
PRISMA 2020 flow diagram for identified studies

Our initial search yielded a total number of 7.063 articles. To refine the dataset, we first utilized the SR Accelerator to remove any duplicates, which reduced the total to 6.021 (Fig. 1) [21]. Included papers were managed using EndNote (Version 21.5, Clarivate Analytics). All stages of study selection, data abstraction, and quality assessment were carried out independently by two reviewers (J.K. and N.J.). Any disagreements were resolved by consulting a third reviewer (H.N.).

This preliminary screening revealed a trend: while a substantial number of articles focused solely on artificial intelligence or esophagectomy, only a few addressed both topics. Many articles exclusively examined AI applications unrelated to esophagectomy, while others focused on esophagectomy without mentioning AI. With this process, 27 articles remained relevant based on their titles.

Next, we reviewed the abstracts of the 27 remaining articles. Studies focusing on risk prediction, diagnostics, or animal models were excluded, as they were considered beyond the scope of this review. Excluded studies are listed in **Supplement 2**.

A total number of six articles aligned with our scope and objective, addressing the use of AI during the intraoperative setting.

### Risk-of-bias assessment

Two reviewers (J.K. & N.J.) assessed the included studies using Version 2 of the Risk of Bias in Non-randomized Studies of Interventions (ROBINS-I V2) assessment tool [22]. For visualization, the Risk-of-bias VISualization (robvis) tool was utilized [23].

### AI in context of this review

The terminology of ML, DL, Convolutional Neural Networks (CNN), transfer learning (TL), and active learning (AL) as used in this manuscript is shown in Table 1.

**Table 1:** Terminology of Artificial Intelligence

Form of AI	Definition
Artificial Intelligence (AI)	AI refers to the simulation of human intelligence in machines, enabling them to perform tasks that typically require human cognition
Machine Learning (ML)	ML is a subset of AI that uses algorithms to allow computers to learn from and make predictions based on data
Deep Learning (DL)	DL is a further subset of ML that uses neural networks with multiple layers to model complex patterns in data
Convolutional Neural Networks (CNN)	CNNs are specialized deep learning models designed for image processing, using convolutional layers to detect patterns
Transfer Learning (TL)	TL involves reusing a pretrained model on a new but related problem to improve learning efficiency and accuracy
Active Learning (AL)	AL is a technique in ML where the model selects the most informative data points for labeling to improve training efficiency

### Study aim

The primary objective of this study was to analyze the extant literature on the implementation of artificial intelligence in esophagectomy procedures with focus on the study design, annotation effort, and implementation of AI. Second, the potential future applications of the technology were evaluated in terms of their broader field of use.

## Results

To ensure clarity, the findings of this review were organized into thematic categories, reflecting the specific AI application in esophagectomy. These categories include anatomy recognition, instrument recognition, phase recognition, annotation, and implementation of artificial intelligence (Table 2).

**Table 2:** Design of the seven selected studies

Study author & year	AI model	Training datasets (videos/frames)	Evaluation metrics	Main application area/task type
Brandenburg et al. (2023)	Bayesian ResNet18	26 videos/14,004 frames	F1-Score, Precision, Recall, McNemar Test	Anatomy & Instrument Recognition/Image-level classification
Den Boer et al. (2023)	CNN (U-Net-like architecture)	200 videos/1050 frames	Dice Score, Hausdorff Distance, Pixel Accuracy	Anatomy Recognition/Semantic segmentation
Furube et al. (2024)	DeepLab v3 Plus	120 videos/50 frames per RLN	IoU (Intersection over Union)	Anatomy Recognition/Semantic segmentation
Sato et al. (2022)	DeepLabv3 +	28 videos/3000 frames	Dice Score	Anatomy Recognition/Semantic segmentation
Eckhoff et al. (2023)	TEsoNet (CNN + LSTM)	40 videos	Accuracy	Phase Recognition/Image-level classification
Takeuchi et al. (2022)	TeCNO (Temporal Convolutional Network)	31 videos	Accuracy, Precision, Recall, Confusion Matrix	Phase Recognition/Image-level classification

### Risk-of-bias assessment in the included studies

**Fig. 2:**
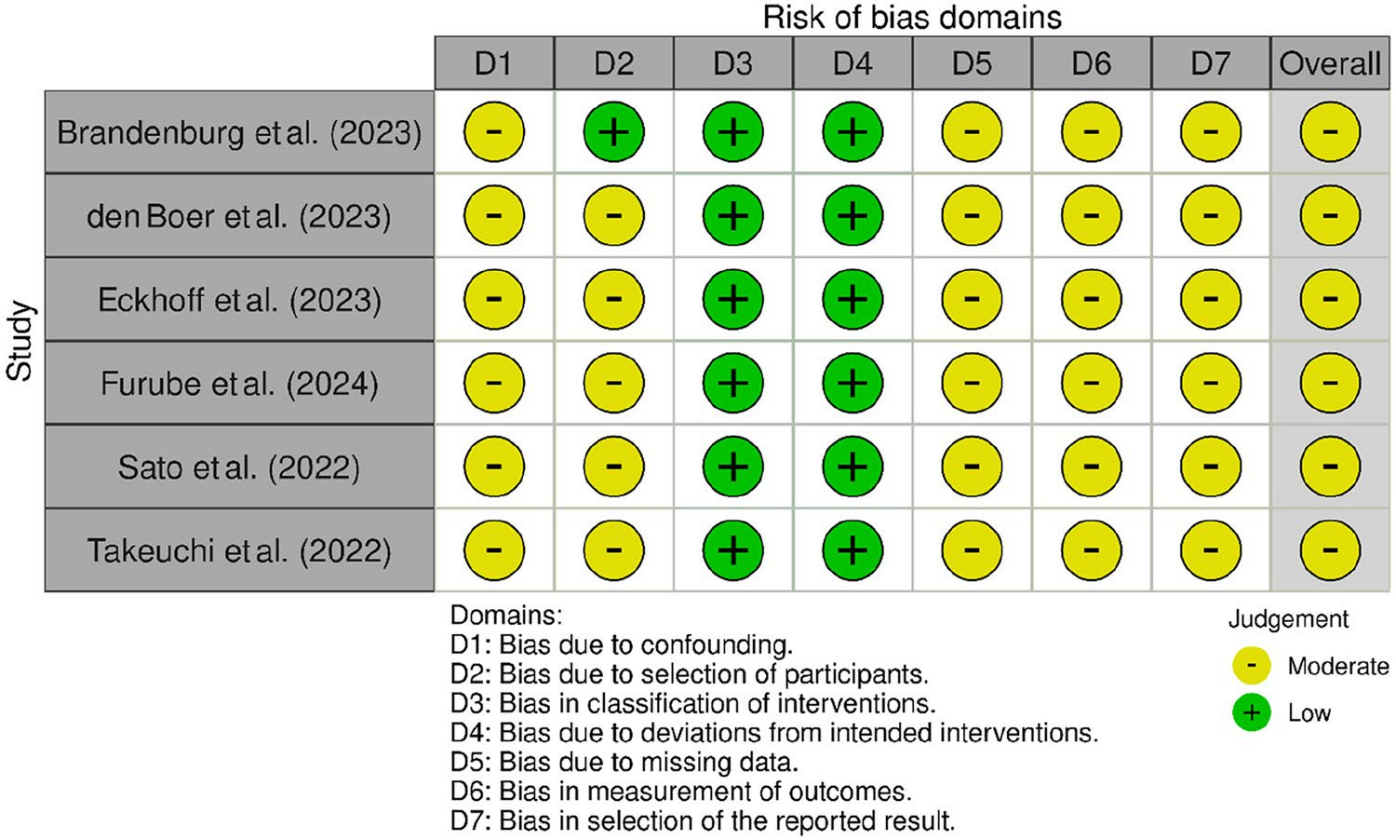
Risk-of-bias assessment of included studies [23]

The six included studies were deemed to be at moderate risk of bias (Fig. 2). Risk of bias for classification of intervention and deviation from intended intervention was judged to be generally low. Areas of risk of bias were primarily related to limited information on confounding, selection of participants, and handling of missing data. Furthermore, risk of bias in measurement of outcomes and reporting was assessed to be moderate to severe.

Due to the limited number of included studies (n = 6), a formal assessment of publication bias (e.g., funnel plots or statistical tests) was not feasible. Reporting of favorable outcomes in all six studies raises concern that negative or null findings remain unpublished.

### Study design, annotation efforts, and implementation of AI

Annotation was an essential preliminary step in all six studies, serving to train the AI model effectively. Each study adopted unique strategies and measures to ensure quality and reliability of annotations. All six studies investigated the application of AI in MIE or RAMIE, centering on three primary use cases: anatomical structure recognition, instrument recognition, and phase recognition. The AI models mostly included deep learning architectures, such as CNNs, Bayesian neural networks, and temporal convolutional networks (TCNs).

Brandenburg et al. analyzed 26 RAMIE videos, with the first two videos providing a start set of 344 equidistantly selected frames (sampled every two minutes). The remaining videos were divided into 20 training videos and four test videos, resulting in a test set of 604 frames for performance evaluation, which were annotated by six medical experts independently. The task was formulated as image-level classification, as the models were trained to detect the presence of binary features or to assign ordinal levels to graded features, not to delineate pixel-level boundaries [24]. For statistical analysis, inter-rater-agreement was applied using the kappa-score. Afterward, they introduced a Bayesian ResNet18 model—an ML-based algorithm chosen for its ability to predict uncertainty through Monte Carlo dropout—to detect the gastric tube and azygos vein in RAMIE videos. They then extended the study to incorporate “Surgomic features”, encompassing bleeding and smoke levels, anatomical structures (gastric tube, azygos vein), and surgical instruments. Their aim was to minimize manual annotation effort while maintaining strong model performance by incorporating AL, to select decisive frames from surgical videos. They then compared AL to Equidistant Sampling (EQS). The model was first pretrained on ImageNet, before being fine-tuned on surgical video data, establishing a baseline for further adaptation on the RAMIE data set [25].

Den Boer et al. assigned the annotation process to a Ph.D. candidate, with complex and random frames revised in collaboration with an expert upper gastrointestinal surgeon during two feedback sessions. Their dataset comprised over 200 RAMIE procedures, yielding 1050 frames divided into an 850-frame training set and a 200-frame test set. A real-time anatomy recognition model was developed, consisting of a semantic segmentation model with a U-net-like architecture, using EfficientNet-B0 as the encoder, chosen for its high performance and relatively small parameter size, making real-time segmentation during surgery feasible. Three pretraining strategies were explored: 1. training from scratch with random weight initialization, 2. pretraining on ImageNet, and 3. pretraining on GastroNet. Model training used cross-validation with up to 1000 epochs with early stopping.

Dice coefficient, 95% Hausdorff distance, and pixel-level accuracy were used as primary evaluation metrics [11].

Eckhoff et al. relied on surgical residents at the postgraduate year 3 level and above for annotations. A panel of three board-certified upper gastrointestinal surgeons defined clinically relevant and computer vision-compatible phase definitions for laparoscopic procedures. Annotation was performed using the Anvil Video Annotation Research Tool for sleeve gastrectomy cases and .csv files for esophagectomy cases. Surgical videos from 40 cases of the laparoscopic part of IL-Esophagectomy were used. They focused on knowledge transfer for phase recognition using a model called TEsoNet. TEsoNet combined a CNN and an LSTM (Long Short‑Term Memory) based network, to recognize surgical phases in Ivor-Lewis (IL) esophagectomy. This task represents image-level classification, as the model assigns each video segment to one of several predefined operative phases rather than producing pixel-level delineations [24]. A larger source dataset of sleeve gastrectomy procedures (80 videos) was used to pretrain their network and then fine-tune it on the smaller IL dataset (40 videos). Accuracy served as the main metric of evaluation, capturing the ratio of correctly identified phases [26].

Furube et al. engaged a total of three annotators, including two certified surgeons for the initial annotations and one expert surgeon for final confirmation. They used 120 RAMIE videos of their own institution for training and 8 videos from another institution for testing. The test set included 50 frames each for the right recurrent laryngeal nerve and the left recurrent laryngeal nerve.

They sought to create an AI model for detecting and segmenting the right and left recurrent laryngeal nerve (RLN), subclavian artery, and trachea during RAMIE. The authors applied DeepLab v3 Plus, a deep learning model, for sematic segmentation at pixel level. Training was conducted on an annotated dataset of 120 RAMIE videos and testing was conducted on a separate annotated dataset of 8 RAMIE videos.

Model performance was examined with Intersection over Union (IoU) [27].

Sato et al. employed two annotators, a general surgeon with experience in fewer than 20 thoracoscopic subtotal esophagectomies and an expert surgeon with experience in over 100 cases. The general surgeon labeled the recurrent laryngeal nerve (RLN) in thoracoscopic esophagectomy images, which were subsequently reviewed and assessed by the expert surgeon. Annotation reliability was evaluated using the Dice coefficient. Their dataset consisted of 28 MIE videos, with 2700 frames used for training, 300 frames for validation, and 40 frames extracted from the remaining 8 videos for the test set (5 frames per video).

They implemented a DeepLab v3 + CNN to detect the recurrent laryngeal nerve (RLN) in thoracoscopic esophagectomy videos. This corresponds to semantic segmentation, as the model assigns class labels at the pixel level rather than classifying whole images or detecting instances [24]. The authors fine-tuned the model, that had been pretrained on the 2012 PASCAL Visual Object Classes (VOC) Challenges dataset, on the annotated images. Model performance was assessed using the Dice coefficient [28].

Takeuchi et al. performed video annotations manually and independently by two board-certified general surgeons. Discrepancies were resolved through discussion to achieve consensus. The authors used 31 videos of patients undergoing RAMIE as primary treatment for esophageal cancer.

They focused on automated phase recognition in RAMIE using a multi-stage temporal convolutional network (MS-TCN) named TeCNO. Image-level classification was applied, as the model assigns each video frame to one of several predefined surgical phases rather than producing pixel-level delineations [24]. They defined nine critical surgical phases and trained the model on 31 annotated videos from RAMIE procedures using fourfold cross-validation and accuracy served as the principal metric.

Another part of the study was to explore potential applications for evaluating surgical proficiency. A comparison was drawn between phase durations in an early-versus-late-period group using the Mann–Whitney U test [29].

### Anatomy recognition

Four of the six studies explored the application of AI in identifying anatomical structures during esophageal surgery, demonstrating its potential in anatomy recognition. Targeted structures included solid organs (e.g., gastric tube, trachea, right lung), vessels (e.g., azygos vein, vena cava, aorta, subclavian artery), and nerves (e.g., recurrent laryngeal nerve).

Brandenburg et al. concentrated on identifying the gastric tube and azygos vein. Anatomical structure recognition was less accurate, with the blood feature performing lowest (F1-score 0.47). Furthermore, no significant difference was observed between AL and EQS for anatomical structure recognition [25].

Den Boer et al. investigated the recognition and localization of the azygos vein, vena cava, aorta, and right lung. Pretraining on ImageNet and GastroNet outperformed training from scratch, especially for more challenging segmentations like the aorta (Wilcoxon signed-rank test, p < 0.05). The highest median Dice score was observed for the vena cava (0.79) and right lung (0.89). Moreover, the model’s real-time performance of 39 frames per second underlines its feasibility for interoperative use [11].

Furube et al. focused on recognizing the recurrent laryngeal nerve, the subclavian artery and the trachea. IoU was 0.40 ± 0.26 for the right RLN, 0.34 ± 0.27 for the left RLN, and 0.60 ± 0.33 for the trachea. While these results left room for improvement, surgeons viewing AI-labeled frames recognized RLNs earlier and more accurately than those without AI labeling [27].

Sato et al. emphasized localizing the RLN. They reached a Dice coefficient of 0.58, close to that of expert esophageal surgeons (0.62) and significantly higher than the general surgeons (0.47, *p* = 0.0019) [28].

### Instrument recognition

Recognizing instruments was explored in one out of the six studies reviewed. This study aimed to identify various surgical instruments during operations.

Brandenburg et al. focused on identifying six specific instruments: the Vessel Sealer, Metal Clips, Cautery Hook, Scissors, Large Clip Applier and Suction Device. The authors found that instrument detection showed the highest performance, particularly for the permanent cautery hook (F1-score 0.95, Precision 0.96, Recall 0.94), while according to McNemar Test, AL outperformed EQS for instrument recognition (*p*  < 0.05) [25].

### Phase recognition

Two of the six studies included in this review investigated phase recognition during esophageal surgery.

Eckhoff et al. explored phase recognition in the laparoscopic part of IL Esophagectomy. They decided on, in their opinion, critical phases in the operation being, Port Placement, Liver Retraction, Dissection of the Gastrohepatic Ligament, Clipping and Division of the Left Gastric Artery, Hiatal Dissection, Dissection of the Gastrocolic Ligament, ICG perfusion check, Formation of the Gastric Conduit, Mediastinal Dissection, and Final Inspection. When trained and tested on sleeve gastrectomy, an accuracy of 87.7% ± 7% was achieved. On esophagectomy while training on a small dataset (5 videos) initial accuracy was 36.3% ± 30.7%, improving to 49.7% ± 29.8% with 30 training videos. Combining sleeve gastrectomy and esophagectomy training data, the overall accuracy improved to 40.8% ± 32.6% [26].

Takeuchi et al. worked on phase recognition as well, splitting the procedure in phase 1 (preparation), phase 2 (lower mediastinal dissection), phase 3 (upper mediastinal dissection), phase 4 (division of the azygos vein), phase 5 (subcarinal LND), phase 6 (right RLN LND), phase 7 (left RLN LND), phase 8 (transection of the esophagus), and phase 9 (post-dissection to completion of surgery). The overall accuracy was 84% and results showed that the AI-based phase durations for preparation (*p*  = 0.012), post-dissection to completion (*p*  = 0.003) and no step (*p* < 0.001) significantly differed between the two groups (early-period versus late-period), consistent with the ground truth [29].

A detailed summary of dataset characteristics and reported performance metrics across the included studies is provided in Table 3, facilitating cross-study comparison despite heterogeneous aims and evaluation methods.

**Table 3:** Summary of dataset characteristics and reported performance metrics

Study	Task	Performance metrics
Takeuchi et al. (2022)	Surgical phase recognition	Accuracy: 84%; precision (overall): ~ 0.84; per-phase recall: 58–93%
Eckhoff et al. (2023)	Surgical phase recognition (CNN + LSTM, TL)	Accuracy depending on setup: 87.7% ± 7% (sleeve gastrectomy), 36.3% ± 30.7% (5 RAMIE videos), 49.7% ± 29.8% (30 RAMIE videos), 24–36% with transfer only; 40.8% ± 32.6% (co-training)
Sato et al. (2022)	RLN segmentation	Dice coefficient: 0.58 (AI), 0.62 (expert surgeons), 0.47 (general surgeons); AI vs. experts: n.s. (*p* = 0.26); AI vs. general surgeons: * p* = 0.019
Furube et al. (2024)	RLN segmentation	AUC: 0.92 (left RLN), 0.88 (right RLN); Dice: 0.72; Sensitivity: 0.86; Specificity: 0.89; IoU: 0.40 ± 0.26 (right), 0.34 ± 0.27 (left); Surgeon recognition at start of LND with AI: 81.3% vs. 46.9% without AI (* p* = 0.004); IoU during LND with AI: 0.59 ± 0.18 vs. 0.40 ± 0.29 (* p* = 0.010)
den Boer et al. (2023)	Anatomy recognition (Bayesian NN)	Baseline Dice: 0.64; With GastroNet pretraining: 0.75 (* p* < 0.05). Median Dice (algorithm): 0.79 (azygos vein/vena cava), 0.74 (aorta), 0.89 (lung). Compared to expert annotations: 0.70 (vena cava/azygos), 0.88 (aorta), 0.90 (lung). Inference time: 0.026 s (39 Hz)
Brandenburg et al. (2023)	Surgomic feature recognition (Active Learning)	Mean F1: 0.75 ± 0.16 (all features); F1 instruments: 0.80 ± 0.17; κ > 0.82 (inter-rater). Compared to EQS, AL achieved significantly better instrument recognition (McNemar test), selected more frames of rare instruments (1512 vs. 607), reached higher F1-scores for common instruments, and required fewer training frames

## Discussion

This systematic review explores the emerging yet still very limited research on AI in esophageal surgery. Despite AI’s rapid advancements across various surgical fields, its application in esophagectomy remains scarce. Our initial literature search identified over 7.000 articles, yet only six articles met our inclusion criteria, underscoring the limited focus on this topic. It is worth mentioning that Cizmic et al. published a review on this topic [30]. However, they did not conduct a systematic search and their review did not include the works of Eckhoff, Furube, and Sato.

As summarized in Table 3, performance across studies varied widely depending on the target task and dataset size. While instrument recognition consistently achieved the highest scores (e.g., mean F1 up to 0.95), anatomical recognition of fine structures such as the RLN remained considerably more challenging, with loU and Dice values reflecting substantial variability across studies.

A major focus in the studies was the implementation of AI to enhance recognition of anatomical structures, surgical instruments, and procedural phases. While the studies showed promising results, particularly in instrument recognition (e.g., cautery hook, F1: 0.95), performance in other areas remained inconsistent. For instance, anatomy recognition of the RLN (IoU: 0.40) and blood (F1: 0.47) showed large variability [25, 27]. It is noteworthy that AI models can exhibit a level of proficiency comparable to that of expert surgeons, particularly in tasks such as the identification of the RLN, which has been identified as a challenging aspect of surgical detection, even for general surgeons [28]. Takeuchi et al. reported a highly accurate surgical phase recognition system with an accuracy of 84%, whereas Eckhoff et al. described a more moderate performance. However, they suggested a TL approach by leveraging data from high-volume procedures like laparoscopic cholecystectomy to mitigate data limitations in low-volume procedures like RAMIE [26, 29]. Interestingly, Eckhoff et al. combined a CNN for image recognition with an LSTM for sequential data recognition.

Different AI models were deployed, including CNNs, U-Net-based architectures, Bayesian neural networks, and temporal convolutional networks (e.g., MS-TCN), to analyze surgical videos in real time, reflecting AI’s role in surgical settings. However, implementing these models requires extensive computational power and large-scale annotated datasets, presenting a significant challenge for low-volume procedures like esophagectomy, where data are inherently limited compared to high-volume surgeries. One potential strategy to address those challenges is the application of AL, which can reduce the total annotation burden by iteratively selecting the most informative frames for expert annotation [25]. AL has shown promising results, particularly in identifying commonly used instruments, but remains limited in detecting rare events, such as bleeding or smoke, highlighting the need for further improvement in this area. Beyond AL, several studies have explored alternative approaches, such as pretraining models on large-scale databases like ImageNet and specialized gastroenterological datasets like GastroNet. This approach led to significant improvements in anatomical structure segmentation (p < 0.05) [11].

An evaluation of the studies included in the analysis was conducted in accordance with the NASA technology readiness levels (TRL) [31]. The majority of the studies were found to be at levels 4 or 5, as per the TRL classification system. However, it should be noted that Brandenburg et al. reported a demonstration in a real-time intraoperative setting, though no results were included in their report.

### Subgroup analysis

Marked differences in recognition performance across anatomical structures underscore the importance of task-specific analysis. For instance, the vena cava in den Boer et al. achieved a median Dice of 0.79, whereas the left RLN in Furube et al. achieved an IoU of only 0.34. These discrepancies can be explained by clinical realities: large, fixed structures with clear image features (aorta, vena cava) are easier to detect, whereas small, variable structures (RLN) remain challenging. Improving recognition of such difficult targets will likely require multimodal integration, refined model architectures and larger, more diverse training sets. While detection of larger structures is important to create and improve AI models, reliable recognition of subtle structures such as the RLN is particularly relevant, as failure in recognition directly increases surgical risk.

### Heterogeneity analysis

The included studies exhibited substantial heterogeneity across several dimensions, affecting comparability and reliability. The surgical approach varied, with some analyzing MIE like Takeuchi et al. and Sato et al. and the others RAMIE, each associated with distinct workflows and visual perspectives. Further, annotator qualification differed considerably, ranging from medical student and resident to expert surgeon. This introduced variability in annotation quality and increased the risk of measurement bias. At last, performance indicators were reported inconsistently, with some using pixel-level segmentation metrics (Dice, IoU), like Furube et al. and den Boer et al., and others relied on image-level classification measures (accuracy, F1) as seen in Takeuchi et al., Eckhoff et al., Brandenburg et al., and Sato et al. Overall, these differences complicate cross-study benchmarking. Therefore, future work must focus on standardized datasets, harmonized annotation protocols, and consensus on reporting metrics to reduce heterogeneity and strengthen reproducibility.

### Bias considerations

The risk of bias across studies was generally moderate but unevenly distributed. Measurement bias, for example, arose from inconsistent definitions of "accuracy"—pixel-level segmentation in some studies versus image-level classification in others—limiting direct comparability. Moreover, several studies failed to report how annotators were trained or whether annotation consistency was valid, raising concerns about ground-truth reliability. Selection bias is also relevant, as datasets were mostly institution-specific and may not represent broader surgical population. To mitigate these risks, future studies should implement standardized operating procedures for annotation, as mentioned in de Backer et al., mandate reporting of annotator training, and ensure external expert validation of datasets [32].

### Future directions

For future studies, collaboration among specialized centers is essential to assemble large and diverse multicenter datasets. Initiatives such as the TIGER-SQA study illustrate how structured video collection and centralized quality assessment can provide standardized benchmarks and lay the foundation for surgical AI [33]. Annotation pipelines should be streamlined and tied to explicit ground truth to reduce subjectivity. Recent multicenter experiences have shown that hierarchical, team-based annotation—combining trained medical students, lay annotators, and expert validation layers—can both increase efficiency and maintain accuracy [32]. Embedding such frameworks into future AI initiatives in esophageal surgery would reduce burden, improve scalability, and ensure robustness across institutions. Furthermore, projects should streamline annotation protocols and incorporate advanced semi-supervised learning techniques, building active learning strategies to reduce the need for time-intensive manual labeling. This approach could accelerate model training, while improving precision, ultimately making AI development more efficient and scalable [25]. AI models rely on large, labeled datasets, especially in supervised learning. This poses a problem due to the low volume of esophagectomies compared to more commonly performed procedures creating a major bottleneck. Several studies have investigated potential solutions, including hierarchical annotation approaches and advanced sampling methods. Nevertheless, further advancements in automated or semi-automated annotation techniques are still needed to facilitate AI implementation in surgical applications [25, 32].

In the long run, AI-driven support may play a crucial role in intraoperative safety, for example by highlighting critical structures or flagging potential errors in real time. To achieve this, appropriate performance metrics for surgical cases must be adopted, ideally within standardized frameworks such as the Metric Reloaded initiative [24].

Moving AI-powered tools from proof-of-concept studies into the operating room requires overcoming significant hurdles. Key barriers include technical integration into complex OR ecosystems, ensuring clinician trust and acceptance and navigating the regulatory landscape.

A fundamental prerequisite for effective AI assistance, particularly for integration with robotic platforms, is fine-granular surgical workflow recognition. For a collaborative robot to become a context-aware partner, it must comprehend the procedure with human-like detail, understanding the interplay of actor, instrument, action, and target [34]. This requires that the AI model’s performance is compatible with the real-time demands of surgery. While instantaneous processing is ideal, a latency of around 1 s is likely tolerable, mimicking the natural reaction time of a human assistant and representing a realistic initial goal for system developers. Achieving this level of integration necessitates adherence to interoperability standards like IEEE 11073 SDC to ensure seamless data exchange between the AI system, robotic platforms (such as the da Vinci system), and other OR devices [35].

Clinician trust and acceptance are critical issues when attempting to bring AI into the operating room. In a survey, interprofessional OR staff expressed significant concerns regarding loss of psychological safety, the potential for the data to be used for surveillance, and issues of privacy [36]. Overcoming this requires a transparent implementation strategy that frames the technology as a tool for learning, quality improvement, and enhancing patient safety, rather than for scrutiny. Engaging all stakeholders from the outset is crucial for building a constructive culture and ensuring buy-in. Furthermore, moving away from "black box" models toward systems that are transparent and explainable can demystify AI-driven recommendations and increase clinical confidence [37].

Finally, any AI tool intended for clinical use must navigate a rigorous regulatory and liability framework. These tools are often classified as "Software as a Medical Device" (SaMD) and are subject to approval from bodies like the U.S. FDA or conform to the EU’s Medical Device Regulation (MDR). This process demands extensive documentation, formal risk management, and high-quality external validation studies that far exceed the scope of typical academic research. This also raises complex questions of liability: if an AI-guided decision leads to a poor outcome, who is responsible? Establishing clear regulations around liability and defining the technology’s Level of Autonomy (LoA) are essential steps that must involve surgeons, manufacturers, and regulators to ensure that patient safety is never compromised [34]. Furthermore, beyond technical compatibility, strict privacy regulations (e.g., GDRP, HIPAA) and heterogeneous international standards complicate data sharing and algorithm validation [38].

A realistic path forward involves a staged approach. Instead of aiming immediately for complex, high-autonomy functions, development should focus first on "low-hanging fruit”, which are applications that offer high clinical value with lower risk. These could include descriptive tools that provide surgeons with data-driven insights rather than prescriptive commands, or automated systems for surgical documentation and reporting, which would reduce administrative burden without directly influencing surgical action.

### Limitations

This review has several limitations. First, only six studies met the inclusion criteria, limiting generalizability and preventing meta-analysis due to heterogeneity. Second, the small, mostly single-institution datasets raise concerns of overfitting and restrict external validity. The six studies included in this review differed significantly in their objectives, methodologies, and evaluation metrics. This limitation restricts statistical power and makes cross-study comparison difficult. Third, annotation subjectivity continues to pose a challenge, as ground-truth labels were often provided by small groups without independent external validation. Furthermore, differences in surgical setup, data sampling rates, and annotation strategies make reproducibility challenging. Fourth, all studies reported positive outcomes, raising the possibility of publication bias. This could overestimate AI’s true effectiveness in esophageal surgery. This limitation should be taken into account, when interpreting feasibility and performance results. Finally, broader challenges, such as regulatory approval, data protection laws, and clinician acceptance, must be recognized as key hurdles on the path to clinical translation.

## Conclusion

While Al in esophageal surgery is still in its early stages, the included studies provide proof-of-concept evidence for its role in anatomically complex procedures. To fully harness Al’s potential, research must move from small, heterogeneous, single-center efforts toward coordinated, large-scale, multi-institutional initiatives with standardized protocols, robust validation, and clear clinical integration strategies.

## Conflict of interest

The authors declare no competing interests.

## Supplementary Information

Below is the link to the electronic supplementary material.

Supplementary file1 (DOCX 17 KB)

## Data Availability

No datasets were generated or analyzed during the current study.
