# Supplementary material for: Application of artificial intelligence in esophageal surgery: a systematic review
Source: J Robot Surg. 2025 Oct 16;19(1):694. doi: 10.1007/s11701-025-02854-9 (PMC12532729; doi:10.1007/s11701-025-02854-9)
Supplement: Supplementary file 1 — Supplementary file1 (DOCX 17 KB) [file 11701_2025_2854_MOESM1_ESM.docx]

Supplement 1: Search terms

("Esophagectomy"[MeSH] OR "Esophagus"[MeSH] OR "Esophag* Surgery" OR esophagectom* OR oesophagectom* OR "Ivor Lewis" OR "Esophag* resect*" OR RAMIE OR "Robot-assisted minimally invasive esophagectomy" OR MIE OR "Minimally invasive esophagectomy") AND ("Artificial Intelligence"[MeSH] OR "Machine Learning"[MeSH] OR artificial intel* OR machine learn* OR AI OR "AI model*" OR "Deep learn*" OR algorithm* OR autonom* OR comput* OR anatom* recogn* OR anatom* detect* OR "Phase* recogn*" OR "Phase* detect*" OR "Pattern recogn*" OR "Pattern detect*" OR "Video* recogn*" OR "Video* detect*" OR "Neural Network*" OR "Convolutional neural network*" OR CNN OR TCN OR "Temporal Convolutional Network" OR RNN OR "Recurrent Neural Network" OR TeCNO OR "Computer Vision" OR "Augment* reality" OR "Computer-Assisted Diagnosis" OR "Predic* model" OR "Transfer learn*")

Supplement 2: Excluded studies in abstract and full-text screening

| **Authors** | **Year** | **Title** | **Exclusion criteria** |
| --- | --- | --- | --- |
| Bolourani et al. | 2021 | Using machine learning to predict early readmission following esophagectomy | Risk prediction |
| Chen et al. | 2020 | Prediction of Lymph Node Metastasis in Superficial Esophageal Cancer Using a Pattern Recognition Neural Network | Risk prediction |
| Ghareeb et al. | 2022 | Integrating the Hill's Classification of Gastroesophageal Junction into the Artificial Intelligence Predictive Model for Gastroesophageal Reflux Disease after Sleeve Gastrectomy; a Room for Improvement? | Risk prediction |
| Jung et al. | 2023 | Prediction of postoperative complications after oesophagectomy using machine-learning methods | Risk prediction |
| Klontzas et al. | 2024 | Prediction of Anastomotic Leakage in Esophageal Cancer Surgery: A Multimodal Machine Learning Model Integrating Imaging and Clinical Data | Risk prediction |
| Li et al. | 2022 | Machine learning models predict lymph node metastasis in patients with stage T1-T2 esophageal squamous cell carcinoma | Risk prediction |
| Liu et al. | 2020 | An artificial neural network model predicting pathologic nodal metastases in clinical stage I-II esophageal squamous cell carcinoma patients | Risk prediction |
| Perez Quintero et al. | 2024 | PROFUGO study protocol: Predictive model for the early diagnosis of anastomotic leak after esophagectomy and gastrectomy | Risk prediction |
| Rahman et al. | 2020 | Machine learning to predict early recurrence after oesophageal cancer surgery | Risk prediction |
| Rahman et al. | 2023 | The AUGIS Survival Predictor: Prediction of Long-Term and Conditional Survival After Esophagectomy Using Random Survival Forests | Risk prediction |
| Sun et al. | 2022 | Deep Learning-based Risk Prediction Model for Postoperative Healthcare-associated Infections] | Risk prediction |
| Van de Beld et al. | 2024 | Complication Prediction after Esophagectomy with Machine Learning | Risk prediction |
| Wang et al. | 2021 | Establishing a survival prediction model for esophageal squamous cell carcinoma based on CT and histopathological images | Risk prediction |
| Winter et al. | 2024 | Enhancing Preoperative Outcome Prediction: A Comparative Retrospective Case-Control Study on Machine Learning versus the International Esodata Study Group Risk Model for Predicting 90-Day Mortality in Oncologic Esophagectomy | Risk prediction |
| Zhao et al. | 2021 | Prediction Model of Anastomotic Leakage Among Esophageal Cancer Patients After Receiving an Esophagectomy: Machine Learning Approach | Risk prediction |
| Gehrung et al. | 2021 | Triage-driven diagnosis of Barrett's esophagus for early detection of esophageal adenocarcinoma using deep learning | AI use in diagnostics |
| Iwagami et al. | 2021 | Artificial intelligence for the detection of esophageal and esophagogastric junctional adenocarcinoma | AI use in diagnostics |
| Rice et al. | 2019 | Precision Surgical Therapy for Adenocarcinoma of the Esophagus and Esophagogastric Junction | AI use in diagnostics |
| Nickel et al. | 2025 | Optimization of anastomotic technique and gastric conduit perfusion with hyperspectral imaging and machine learning in an experimental model for minimally invasive esophagectomy | Animal trials |
| De Backer et al. | 2022 | Multicentric exploration of tool annotation in robotic surgery: lessons learned when starting a surgical artificial intelligence project | AI was not used in surgery |
| Lemmens et al. | 2023 | Performance of a consensus-based algorithm for diagnosing anastomotic leak after minimally invasive esophagectomy for esophageal cancer | AI was not used |
